# Prevalence and associated factors of eating disorders in adults with type 2 diabetes: a systematic review

**DOI:** 10.1186/s40337-025-01391-y

**Published:** 2025-09-26

**Authors:** Aycan Celik Esmer, Zahraa Jalal, Ping Guo, Muzeyyen Seckin

**Affiliations:** 1 Faculty of Health Sciences, Internal Medicine Nursing Department, Uşak University, Uşak, Turkey; 2 Department of Pharmacy, School of Pharmacy, School of Health Sciences, College of Medicine and Health, University of Birmingham, Birmingham, UK; 3 Department of Nursing and Midwifery, School of Health Sciences, College of Medicine and Health, University of Birmingham, East Wing, Medical School, Edgbaston, Birmingham, B15 2TT UK

**Keywords:** Diabetes, Night eating disorder, Binge eating disorder, Eating disorders, Prevalence, Systematic review

## Abstract

**Background:**

People with type 2 diabetes (T2DM) have a higher risk of eating disorders, specifically binge eating disorders (BED) and night eating syndrome (NES) which may affect the diabetes management and long-term outcomes of T2DM. There is limited evidence to determine the prevalence and associated factors of this condition for targeted interventions. Our study aimed to systematically synthesise existing evidence exploring the prevalence of eating disorders and associated factors among adults with T2DM.

**Methods:**

This review was pre-registered with PROSPERO (CRD42024587276). A systematic review was undertaken searching Embase, Medline, PsycINFO, Cochrane Central database. The National Institutes of Health Quality Assessment tool for Observational Cohort and Cross-Sectional Studies was used to evaluate the quality of eligible studies. Given the insufficient number of studies assessing the targeted outcomes, a meta-analysis was not attempted. A narrative synthesis was conducted.

**Results:**

Twelve studies were included with cross-sectional studies. BED and NES were the two most common eating disorders in people with T2DM. Point prevalence was 2.5–29.6% for BED and 1.6–8.4% for NES. No data were available on the prevalence of bulimia and anorexia nervosa. Having eating disorders in T2DM was associated with a low level of psychological well-being, greater depression, anxiety symptoms, and high levels of BMI and HbA1c.

**Conclusion:**

There are psychological, physical and social factors associated with to high prevalence of eating disorders in T2DM. The current literature on eating disorders in T2DM is relatively limited, with few studies applying rigorous methods. Further studies are needed for large, high-quality studies that focus on the management, diagnosis, physical and psychosocial effects, and long-term outcomes of eating disorders in adults with T2DM.

**Supplementary Information:**

The online version contains supplementary material available at 10.1186/s40337-025-01391-y.

## Abstract (plain language summary)

Eating disorders are a notable and growing global public health issue. The prevalence of eating disorders among people with type 2 diabetes is rising sharply, underscoring the need to recognize and address eating disorders in individuals with type 2 diabetes mellitus as a significant clinical concern. The general neglect of eating disorders in individuals with type 2 diabetes mellitus may stem from a combination of physical (e.g., gender), psychological (e.g., shame), and social factors (e.g., social interactions). For instance, some individuals may hesitate to disclose their condition due to feelings of shame, while others may struggle with poor emotional health and increased dissatisfaction with their body image. Our findings provide valuable insights that may guide the development of more personalised and effective interventions for individuals affected. We also provide recommendations for future research, such as including larger samples of more diverse populations and more research on anorexia nervosa and bulimia in type 2 diabetes mellitus.

**Supplementary Information:**

The online version contains supplementary material available at 10.1186/s40337-025-01391-y.

## Background

There is a growing prevalence of diabetes globally, with more than 500 million individuals currently living with type 2 diabetes T2DM [1]. By 2030, the number is expected to grow to 643 million, rising to 783 million by 2045. Nearly three out of four adults with diabetes live in low- and middle-income countries [1]. Individuals living with T2DM have a higher risk of health problems, consisting of stroke, heart attack, and kidney disease [2]. T2DM is also linked to an increased risk of psychiatric conditions, like diabetes distress and eating disorders [3, 4]. Individuals with diabetes may develop unhealthy relationships with food, partly due to the intense focus on food-related control, adherence to dietary restrictions, the need for constant management of glucose levels, and anxiety around diabetes-related complications [5]. Concerns about weight gain or body image issues, particularly when insulin use or changes in eating habits are involved, can exacerbate this [6]. In some cases, to control their weight or manage their health, individuals may resort to disordered eating behaviours [7, 8].

Diagnosis of Anorexia Nervosa, Bulimia Nervosa, and Binge Eating Disorder (BED) is based on the Diagnostic and Statistical Manual of Mental Disorders (DSM-5) diagnostic criteria and each was acknowledged as a separate section [9]. Night Eating Syndrome (NES) was formally recognised as a specific disorder under the category of Other Specified Feeding or Eating Disorders (OSFED). NES and BED represent the most common types of eating disorders in people with T2DM [3, 10, 11]. The Diagnostic and Statistical Manual of Mental Disorders (DSM-5) diagnostic criteria describe BED as eating an unusually large quantity of food within a defined period, often associated with a feeling of losing control and significant distress [3, 12]. BED affects approximately 1.5–2.8% of the general population [13], and those with BED are three to six times more prone to be obese compared to people without the disorder [14]. BED affects an estimated 4–47% of bariatric patients [6].

Several challenges impact the screening and diagnosis of BED, ultimately influencing its treatment outcomes. A significant contributor to this gap is the lack of awareness and clinical familiarity with BED among healthcare professionals, which can lead to underdiagnosis and delayed intervention. Moreover, many patients hesitate to report binge eating behaviours, driven by feelings of shame, guilt, and concerns about potential judgment from healthcare professionals. This leads to suboptimal glycaemic control as disordered eating habits such as limiting food intake, overexercising, and purging can cause blood sugar levels to become unstable and erratic [3].

Kenardy, Mensch [15] found that the frequency of binge eating was positively correlated with poor blood glucose control, as measured by HbA1c, even after adjusting for confounding variables such as BMI and exercise level. Furthermore, research indicates that individuals who experience binge eating episodes are diagnosed with type 2 diabetes mellitus (T2DM) at a significantly younger age compared to those without such episodes. A younger age at T2DM diagnosis is associated with an increased risk of adverse cardiovascular outcomes and higher mortality [16]. Therefore, the earlier onset of T2DM among individuals with BED highlights the critical need for routine screening for T2DM in this population, as well as early intervention strategies targeting BED.

NES is characterised by recurrent episodes of excessive food consumption during the night, either after waking up from sleep (nocturnal ingestion) or after the evening meal (evening hyperphagia). Studies examining night eating symptoms in individuals with T2DM have reported prevalence rates ranging from 8.4 to 12.4% [17–19]. This is significant because it indicates that a noticeable portion of people with diabetes—both Type 1 and Type 2—experience symptoms of night eating, which can have a substantial impact on their overall health and diabetes management [6].

Previous studies have shown that the occurrence of disordered eating and eating disorders is increased in individuals with type 1 diabetes [5, 20, 21]. It is well documented that the co-existence of eating disorders and type 1 diabetes is extremely concerning due to the compounded risks it poses, including elevated levels of HbA1c [5]. This can significantly increase the risk of both acute complications (e.g., diabetic ketoacidosis, or DKA) and chronic complications (like neuropathy, retinopathy, and cardiovascular disease) [22–24]. Thus, eating disorders in individuals with type 1 diabetes have increasingly gained attention in both clinical and media discussions [5]. The finding that 93.8% of patients with type 1 diabetes and eating disorders were diagnosed with type 1 diabetes before their eating disorder diagnosis is particularly insightful [25]. It supports the idea that the challenges associated with managing a chronic conditions like type 1 diabetes could be significant contributing factors to the development of eating disorders [26].

The relationship between eating disorders and T2DM is less well understood compared to Type 1 diabetes, and there has been less research into this specific association. Much of the focus historically has been on Type 1 diabetes, likely because it typically presents earlier in life and often involves more direct medical management, including insulin use [6]. However, eating disorders in individuals with T2DM are also a significant concern, though they may be less frequently discussed [11]. Among this population, BED and NES are the predominant types of eating disorders, with reported rates ranging from under 5–25.6% [21, 27]. This condition may negatively affect the metabolic parameters in individuals with T2DM. More severe binge eating behaviours, as measured by scales such as the Binge Eating Scale (BES), are closely linked to suboptimal glycaemic control, as reflected by elevated HbA1c levels [28], resulting in diabetes-related complications including cardiovascular disease [29].

The general neglect of eating disorders in those with T2DM may, in part, be attributed to psychological and social factors such as shame and stigma, which make it difficult for both people with T2DM and healthcare providers to engage in constructive discussions about eating disorders [30]. Thus, this systematic review aimed to evaluate the prevalence of eating disorders, specifically NES and BED, and identify factors associated with eating disorders in adults with T2DM. This review can help healthcare providers better understand the challenges that arise when these conditions occur together. By highlighting the prevalence of eating disorders and the associated factors, healthcare providers can more effectively identify individuals with T2DM who may be vulnerable to disordered eating, enabling early recognition and the provision of appropriate care and support.

## Methods

A systematic review of existing literature was performed to estimate the prevalence of eating disorders in people with T2DM and to identify factors associated with eating disorders among adults with T2DM. For transparency, the protocol of this review was registered with the International Prospective Register of Systematic Reviews (PROSPERO, CRD42024587276). The review was carried out following the Preferred Reporting Items for Systematic Reviews and Meta-Analyses (PRISMA) [31].

### Literature search

A systematic search strategy was conducted across several databases which include the following: EMBASE, MEDLINE, PsycINFO, COCHRANE Central database. Databases were searched without time limitations. Data resources were collected on August 1, 2024. The systematic review included only studies published in English due to limited translation resources. The search utilised a combination of Medical Subject Headings (MeSH), incorporating all related free texts, sub-terms and sub-headings, Boolean operators and truncation. The search employed the following operators and search terms: Type 2 diabet* OR noninsulin depend$ diabet$ AND eating disorder* OR binge eating disorder (Supplementary Table 1). The reference list of included studies was also searched.

### Eligibility criteria

Original studies using quantitative methods that provided an estimate of the prevalence of eating disorders in people with T2DM were included. To be included, studies were required to diagnose eating disorders according to DSM-IV or DSM-5 criteria and to utilize validated questionnaires or structured interview methods.

Exclusion criteria were as follows: (1) Studies including only people with type 1 diabetes, those having any identified mental disorders and/or women who are pregnant or lactating. (2) Studies involving mixed samples that did not provide separate data specifically for adults with T2DM from people with other types of diabetes (such as gestational diabetes and type 1 diabetes). (3) Studies including people aged < 18 years. (4) Literature reviews, review articles, systematic reviews, and abstracts that provide inadequate information for study evaluation.

### Data extraction

Initial screening of titles and abstracts to identify relevant papers was conducted by one reviewer (ACE). Following initial screening, the full texts of relevant papers were accessed and independently examined by ACE and MS. Any disagreements were resolved through discussion among the reviewers (ACE, MS, ZJ, PG). Data extraction was conducted using two standardized, pre-tested templates designed for quantitative studies. Data was extracted by one reviewer (ACE), and the extraction was checked by other reviewers (MS, ZJ, PG). Disagreements were resolved by involving all reviewers in the discussion (ACE, MS, ZJ, PG).

The extracted characteristics included: author, year, study methods, country, study aim, the characteristics of the study sample (for example, diabetes duration, age range, sample size), eating disorders measurement scale, prevalence of eating disorders, and main results.

### Quality assessment of included studies

The quality appraisal of included papers utilised the National Institutes of Health Quality Assessment tool for Observational Cohort and Cross-Sectional Studies [32], which consists of attrition, confounders, data collection, design, selection bias, and blinding. Each study was evaluated and categorised as ‘good’, ‘fair’, or ‘poor’. For example, ‘good’ means low risk of bias, translating to a rating of good quality. ACE and MS independently rated all of the studies, and differences in opinion were resolved through discussion with reviewers (ACE, MS, ZJ, PG).

### Data synthesis

This review included all the data reporting eating disorders among people with T2DM. Due to heterogeneity in the aims, designs, and outcomes of included studies, a meta-analysis was considered inappropriate. Thus, a narrative synthesis guided by the Synthesis Without Meta-Analysis (SWiM) [33] was carried out to describe the results of the included studies. Initial data synthesis was performed by a single reviewer (ACE) and subsequently discussed with the full review team (ACE, MS, ZJ, PG).

## Results

**Fig. 1:**
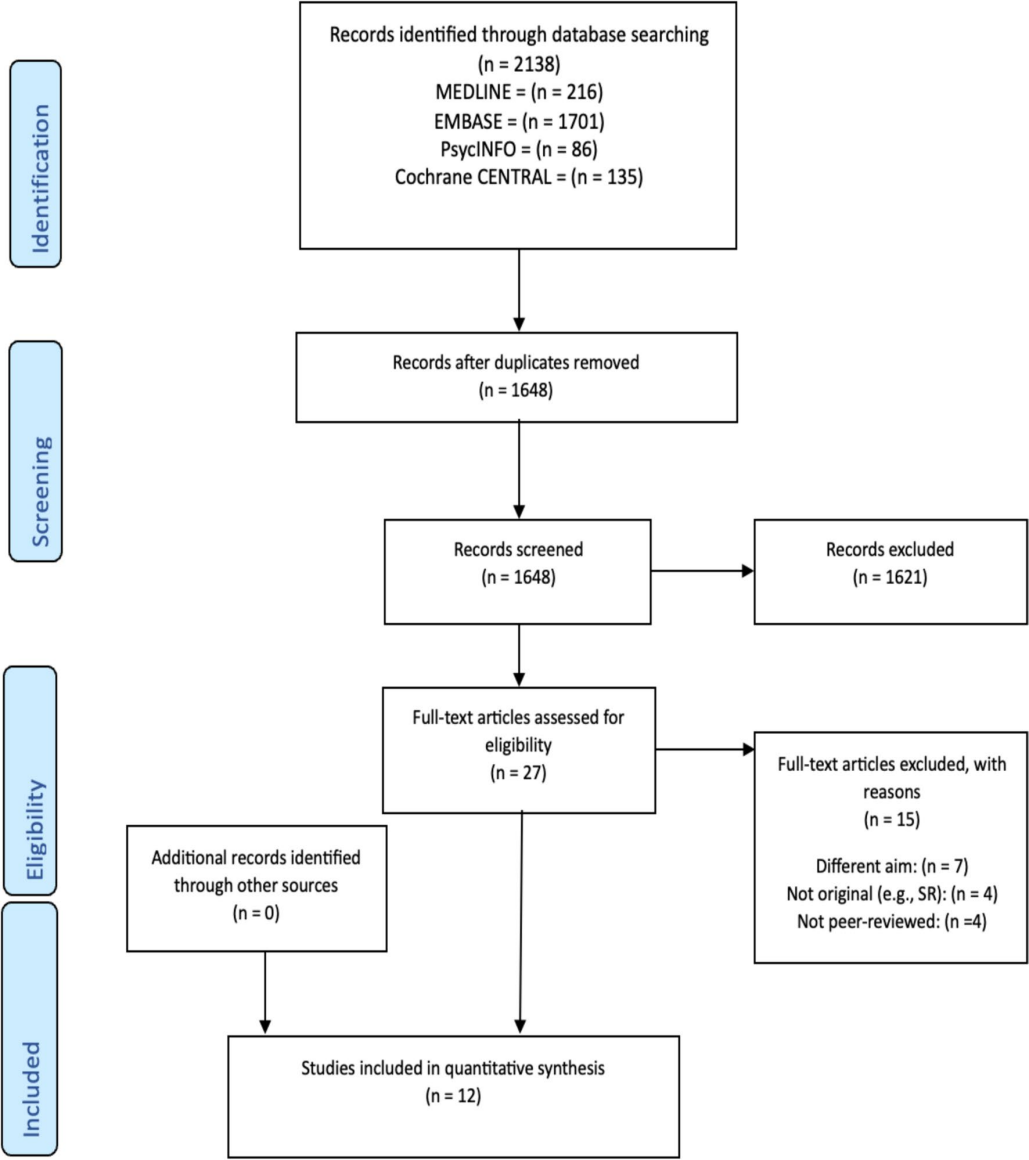
PRISMA flow diagram

A systematic search yielded 2138 papers from the four databases. After removing 490 duplicates, 1648 titles and abstracts were screened based on the eligibility criteria, resulting in exclusion of 1621 papers. Twenty-seven full-text articles were selected to assess for eligibility, and 15 of them were excluded because they did not meet the eligibility criteria. The PRISMA [34] flowchart (Fig. 1) outlines reasons for exclusion in more detail. In total, 12 papers met the inclusion criteria and were included in the systematic review.

### Study characteristics

A total of 12 cross-sectional studies were included. Three of these studies were conducted in the USA, three in India, two in Italy, one in Turkey, Australia, Spain, and Chile. The overall number of people with T2DM who participated in the studies was 4092 people with ranging from a mean age of 48 to 67 years. Studies were published between 2001 and 2023. Inclusion in the review was determined based on the criteria outlined above. The main outcome and objective of all included studies were to examine the prevalence of eating disorders and their association with T2DM. Nine studies reported the prevalence of binge eating disorders (BED) and used different tools. Four studies [27, 35–37] used Eating Attitude Test-26 (EAT-26 scale), a screening measure of eating disorders over past 6 months; Herbozo, Flynn [38] (2015) used Questionnaire of Eating and Weight Patterns-Revised (QEWP-R), assessing the diagnostic criteria for BED over the past 6 months and Eating Disorder Examination-Questionnaire (EDE-Q), measuring the eating disorders over past 4 weeks; Mannucci, Tesi [39] (2002) used EDE-12.0D, a semi-structured interview that provides a profile of psychopathology based on four subscales and a global score over the past 3 months; Nicolau, Simó [27] (2015) used Questionnaire of Eating and Weight Patterns-26 (QEWP-26), assessing diagnostic criteria for the BED over the past 6 months; Allison, Crow [17] (2007) used EDE-Q. Crow, Kendall [40] (2000) used the Impact of weight scale and the Three-Factor Eating Questionnaire (TFEQ), measuring eating behaviours over the past month. Three studies reported the prevalence of night eating syndrome (NES) and used Night Eating Questionnaire (NEQ scale), screening for symptoms of NES over the past 3 months [17, 19, 37]. Two studies reported the prevalence of eating disorders and associated risk factors [8, 41]. Most studies (n = 6) established the diagnosis of BED according to DSM-IV criteria, while three studies used DSM-5. This shows the heterogeneity in the method used to diagnose BED and NES, which affect prevalence estimates. Table 1 presents the characteristics of the included studies in detail.

**Table 1:** Summary of included studies

Author Year Country	Study design	Multi-centre Yes/no	Study aim(s)	Participants (n)	Eating disorder	Criteria	Eating disorder measurement scale	Diagnosis n (%)
Study 1- Allison, Crow [17] 2007 USA	Cross-sectional	Yes	To determine the prevalence of BED and NES among applicants to the Look AHEAD (Action for Health in Diabetes) study	People with T2DM (n = 845)	BED and NES	DSM-IV Clinical assessment	EDE-Q NEQ	N = 47 (%5.6) N = 71 (%8.4)
Study 2- Celik, Kayar [35] 2014 Turkey	Cross-sectional	No	To determine the prevalence of BED in T2DM patients and examine the correlation of BED with level of depression and glycaemic control	People with T2DM (n = 152)	BED	DSM-IV	EAT-40	N = 45 (%29.6)
Study 3- Crow, Kendall [40] 2000 USA	Cross-sectional	No	To examine the rates of BED and other psychopathology in T2DM using a structured psychiatric interview and to examine glycaemic control in BED and non-BED groups	People with T2DM (n = 43)	BED	DSM-IV	TFEQ and Impact of Weight Scale	N = 11 (%25.6)
Study 4- Herbozo, Flynn [38] 2015 Chile	Cross-sectional	No	To examine the prevalence of binge eating and its association with dietary adherence, glycaemic control, and psychological factors among indigenous and nonindigenous T2DM patients in Chile	People with T2DM (n = 387)	BED	DSM-5	QEWP-R and EDE-Q	N = 31 (%8)
Study 5- Hood, Reutrakul [19] 2014 USA	Cross-sectional	No	To assess the prevalence of night eating and associated eating, sleep, and mood components in patients with T2DM	People with T2DM (n = 194)	NES	DSM-5	NEQ	N = 13 (%7)
Study 6- Kenardy, Mensch [15] 2001 Australia	Cross-sectional	No	To investigate the relationship between disordered eating, particularly binge eating, and T2DM in women	People with T2DM (n = 215)	BED	DSM-IV	EDI	N = 3 (%6)
Study 7- Krishnamurthy, Gupta [36] 2020 India	Cross-sectional	No	To evaluate the presence of eating disorders and their association with glycaemic control and metabolic parameters in adult patients with T2DM	People with T2DM (n = 256)	BED	DSM-5	EAT-26 and BES	N = 28 (%10.9)
Study 8- Kumar, Alam [8] 2023 India	Cross-sectional	No	To evaluate the presence of eating disorders and their association with glycaemic control and metabolic parameters in adult patients with T2DM	People with T2DM (n = 145)	Eating disorders	NR	EAT-26	%17.3
Study 9- Mannucci, Tesi [39] 2002 Italy	Cross-sectional	No	To assess the prevalence of eating disorders, and of eating disorder symptoms, in overweight and obese patients with T2DM, compared to non-diabetic obese subject	People with T2DM (n = 146) & without T2DM (n = 240)	BED	DSM-IV	EDE 12.0D	Observed prevalence N = 2, female (%2.5 out of 80)
Study 10- Muley, Deshmane [41] 2024 India	Cross-sectional	No	To identify individuals with T2DM with increased risk of eating disorders as well as look into the patter and socio-demographic risk factors of Eds	People with T2DM (n = 254)	Eating disorders	–	EDE-Q and SCOFF	%35 out of 90
Study 11- Nicolau, Simó [27] 2015 Spain	Cross-section	No	To evaluate the frequency of a positive screening eating disorder, especially BED in people with T2DM	People with T2DM (n = 320)	BED	DSM-IV	EAT-26 and QEWP-R	BED, N = 39 (%12.2) Bulimia nervosa, N = 11 (%3.4)
Study 12- Petroni, Barbanti [37] 2019 Italy	Cross-sectional	Yes	To assess prevalence of dysfunctional eating in people with T2DM in Italy as well as its association with socio-demographic characteristics	People with T2DM (n = 895)	Eating disorder BED NES	NR	EAT-26, BES and NEQ	Eating disorder (n = 121, %13.5) BES (n = 72, %8) NEQ (n = 15, %1.6)

T2DM, type 2 diabetes; BED, binge eating disorder; NES, night eating disorder; EDE-Q, Eating Disorder Examination-Questionnaire; NEQ, Night Eating Questionnaire; BES, Binge Eating Scale; EAT-26, Eating Attitude Test-26; DSM-IV, Diagnostic and Statistical Manual of Mental Disorders, Fourth Edition; DSM-5, Diagnostic and Statistical Manual of Mental Disorders, Fifth Edition; QEWP-R, Questionnaire of Eating and Weight Patterns-Revised; SCOFF, the Sick, Control, One, Fat, Food questionnaire; EDI, Eating Disorder Inventory; TFEQ, The Three-Factor Eating Questionnaire

### Quality assessment

Although the majority of studies had a clearly stated aim, their methodological quality was poor to good (Supplementary Table 2). Twelve studies employed the cross-sectional design, which is prone to two frequent risks of bias components: information and selection. The outcome measures were clearly defined in almost all studies, and in most cases, their reliability and validity were established. The outcome measures in one study [17] were mentioned briefly, with no details regarding their validity. Self-report questionnaires were used across all studies, increasing the possibility of social desirability bias [42]. Assessment and mitigation of bias were poorly reported in the majority of studies. Sample size intentions were not justified or clarified in the studies, seven of which were small [8, 15, 19, 35, 39–41]. Nine studies did not define the participant response rate. The reliability and validity of the exposure measures were not explicitly reported in six studies, which also failed to define these measures clearly.

### Prevalence of eating disorders

The prevalence of BED was reported based on 1898 participants across nine studies. The design of all studies was cross-sectional, and 2 were multi-centre studies. Table 1 presents prevalence data in detail. For BED, the total point prevalence was 2.5 to 29.6%. Data on the overall point prevalence of NES were obtained from 1934 participants from 3 studies, including two multi-centre studies [17, 37]. The prevalence of NES ranged from 1.6 to 8.4%. On the other hand, only one study reported bulimia nervosa with a prevalence of 3.4% (n = 11 out of 320). Kumar, Alam [8] and Muley, Deshmane [41] pointed out the prevalence of all eating disorders as described in questionnaires at 17.3% and 35% respectively. No data were available on the prevalence of bulimia and anorexia nervosa.

### Factors associated with eating disorders

Reported correlates of eating disorders were classified into three potential factors: biological, social, and psychological factors. Table 2 provides a summary of these. They were reported either in a correlation or regression analysis with eating disorder scores as the reference or dependent variable.

**Table 2:** Identified eating disorders factors based on regression and correlation analysis

Factors	Studies											
	1	2	3	4	5	6	7	8	9	10	11	12
Age	R*|										R*|	R*|
BMI	R*|		R*/			R*/			R*/			
HbA1c > 6.5%					R*|			R*/	R*/		R*|	
Gender (Female)		R*/	R*/			R*/			R*/	R*/		R*|
Diabetes duration											R*|	
Age at diabetes diagnosis	R*|			R*/		R*/						
Depression symptoms	R*|	R*/		R*/	R*|	R*/						
Anxiety						R*/						
Emotional wellbeing				R*/		R*/						
Social life			R*/									
Body image dissatisfaction				R*/					R*/			
Sleep disruption					R*|							
Eating plan adherence				R*/								
Self-efficacy						R*/						
Education										R*/		

R, reported; *, significant; /, correlation analysis; |, regression analysis; _, not reported

Biological factors positively associated with eating disorders in T2DM consisted of gender, HbA1c level, diabetes duration, and age at diabetes diagnosis. Study 2 [35] reported that the total EAT score of women with T2DM was 22.77 ± 11.92, while it was 21.45 ± 9.60 in men with T2DM, indicating a statistically important difference (*p* = 0.001), but clinically insignificant (the total EAT score < 30). The BMI levels of women with T2DM were considerably higher than that of men with T2DM (*p* < 0.001). In study 12 [37], after adjusting for confounder factors, multivariable logistic regression analysis showed that younger age and female sex were associated with increased risk of dysfunctional eating (P < 0.001). Logistic regression analysis in study 5 [19] showed that the increased NEQ scores were significantly related to the risk of having HbA1C values greater than 7% (odds ratio (OR) = 1.06 [95% confidence interval (CI) 1.01–1.12], *p* = 0.02). In study 1 [17], logistic regression models revealed that a younger age of diabetes diagnosis was related to an elevated risk of eating disorders (OR = 0.94; 95% CI 0.89, 1.00; *p* = 0.04). Likewise, study 4 [38] demonstrated that the binge-eating group with T2DM reported a younger age at diabetes diagnosis (43.98 years [SD = 12.27]) compared to the non-binge-eating group with T2DM (51.87 years [12.70]). Study 11 [27] reported that T2DM people with criteria for BED had a lower T2DM duration (8.5 ± 6.1 vs 12.1 ± 9.6 years; *p* = 0.002) compared with those without BED, based on a logistic regression analysis.

Psychological characteristics, including depression symptoms, anxiety, emotional well-being, self-efficacy, sleep quality, and body image satisfaction, were reported in studies. A positive correlation in study 2 [35] was reported between depression scores and EAT scores (r = + 0.196, *p* < 0.05). In study 5 [19], a comparison of those meeting NES criteria (n = 13) versus non-NES participants (n = 181; below NEQ cutoff) revealed suboptimal sleep quality (t = − 3.70, *p* < 0.001) and more depressive symptoms (t = − 3.77, *p* < 0.001) among the NES group. Binge eating frequency in Study 4 [38] was significantly associated with lower diet self-efficacy (*p* < 0.05), lower emotional well-being (*p* < 0.05), greater body image dissatisfaction (*p* < 0.01), and less likely to adhere to their eating plan (*p* < 0.003). The logistic regression model in study 10 [41] showed that education was a notable predictor of eating disorders (OD = 1.47, 95% CI 1.00–2.16 and *p* = 0.04).

Only one study (study 3) [40] reported social factors. The study reported a positive correlation between work and binge eating (*p* < 0.001), demonstrating that having work was associated with more severe binge eating activity.

## Discussion

This systematic review demonstrated the prevalence of eating disorders and elucidated the factors leading to the emergence of eating disorders in people with T2DM. Our findings indicate a notable point prevalence increase in BED and NES, ranging from 2.5 to 29.6% and 1.6 to 8.4%, respectively. A systematic review, in agreement with this finding, pointed out that the overall prevalence of BED and NES among people with T2DM was 1.2–8.0% for BED and 3.8–8.4% for NES [10]. Eating problems have been shown to be common in people with T2DM, and thus eating behaviours are a significant component of diabetes management, which is particularly neglected in people with T2DM.

NES and BED were the most prevalent in people with T2DM, however the variation in the prevalence of NES and BED among individuals with T2DM was a noteworthy point, which mirrors the results of the previous review [10]. This kind of variation can stem from a variety of factors, including differences in diagnostic criteria, cultural contexts, and methodologies used across different studies. Some studies may have used more stringent diagnostic tools, while others might have relied on self-reported questionnaires or less precise measures.

Our study found eating disorder behaviours among people with T2DM were associated with physical (e.g., BMI, age), psychological (e.g., distress) and social factors (e.g., shame). The relationship between BED and higher BMI is well-documented in this review and aligns with broader research, including data from the World Health Organization (WHO) from 14 countries (*p* < 0.0001). Individuals with BED often experience cycles of overeating followed by guilt or distress, which can lead to significant weight gain over time [43]. However, because of the methodological designs of the included papers in our systematic review, it is challenging to determine the direction of the association among NES/BED and obesity in people with T2DM, specifically given the biological plausibility of a bidirectional relationship. Therefore, additional longitudinal research and randomized controlled trials (RCTs) are required to better understand the relationship between NES/BED and obesity in T2DM people.

Interestingly, this review found that there was no apparent association between NES and BMI or an increased likelihood of obesity. However, NES was associated with poorer glycemic control due to skipping breakfast, choosing fat- and carbohydrate-rich foods at night, eating large amounts at night, insomnia, and morning anorexia. This suggests that the link between night eating and glycemic control may be more complex. Additional research is needed to explore this relationship both at the syndrome level (i.e., NES) and at the symptom level, by separately examining eating behaviors, sleep disturbances, and mood symptoms—each of which may independently influence glycemic regulation in individuals with diabetes.

The four included studies in this systematic review reported that HbA1c levels illustrated a statistically significant association between BED and NES. Similar results were found in previous studies, which also showed that HbA1c levels were higher in people with eating disorders compared to those without [8, 36]. The findings BED and NES are more prevalent among females than males are supported by Nip, Reboussin [44] who reported 64% of those with T2DM (n = 159). Similar to Nielsen and Vilmar [45], this study pointed out that education was a significant predictor of eating disorders (OD = 1.47, 95% CI 1.00–2.16 and *p* = 0.04).

Importantly, this review indicated that engaging in eating disorder behaviours contributes to poorer emotional well-being, greater body image dissatisfaction, anxiety, and depression symptoms. These findings are consistent with a study of young people with T1DM and T2DM, which indicated that individuals with T2DM and binge eating behaviours had higher levels of depression and anxiety symptoms [46]. Depressive symptoms have been linked with reduced quality of life [47], elevated HbA1c levels [48], increased use of medical services [49], increased risk and severity of long-term diabetes related complications [50], and increased all risk of all-cause mortality [51]. These results have highlighted the importance of developing more robust and holistic interventions for T2DM that deal with eating disorders and psychological well-being [12].

### Recommendations for future research and practice

There is a lack of data regarding psychological and social risk factors of eating disorders. Further empirical attention should be given to the early detection of risk factors of eating disorders, especially psychological and social factors among people with T2DM. To address risk factors, a personalized support system for diabetes care in individuals with T2DM should be established and enhanced, enabling healthcare providers to adopt a comprehensive and holistic approach. Individuals with eating disorders and T2DM require more socially and psychologically sensitive diagnostic tools for early recognition, along with comprehensive physical and psychosocial support strategies. These approaches can help healthcare providers gain a deeper understanding of patients' perspectives, values, and preferences. To enhance health and well-being, there is a need for more evidence-based practices and policies that incorporate socially and psychologically sensitive, multidimensional interventions for individuals with eating disorders and T2DM, particularly those from diverse backgrounds. Healthcare providers should take into account the relationship between psychosocial and psychological risk factors—such as experiences of shame and poor emotional well-being—and adverse diabetes outcomes. Research on bulimia and anorexia nervosa in the context of T2DM is limited, highlighting the need for further studies to deepen our understanding of these conditions within this population.

Further longitudinal research connecting complication severity and quality of life in diabetes care is required to elucidate the barriers, benefits, and factors that may modify outcomes. In addition to quantitative research aiming to illustrate the factors associated with eating disorders among people with T2DM, innovative interventions/strategies are required, particularly for this population, to optimise and sustain overall health and well-being over the long term.

### Strengths and limitations

This is the first systematic review examining the prevalence of eating disorders in T2DM and demonstrates the factors contributing to the emergence of eating disorders among people with T2DM, using PRISMA methods and a comprehensive search strategy. This review’s protocol was registered on the PROSPERO database. The study findings reflected a wide range of populations with T2DM across various countries, including Asia, Europe, and the USA. Relevant studies were identified through searches in four databases.

This systematic review has some limitations. Firstly, all studies included were cross-sectional designs that enable a snapshot of data at a single point in time, so a clear cause-and-effect relationship could not be determined. Secondly, most studies were carried out at a single centre, limiting the generalisability of the results. In addition, most studies did not report ethnicity, which may have played a significant role in the relationship between eating disorders and T2DM. Overall, there has been extremely restricted data available to examine the relationship between other types of eating disorders (e.g. bulimia nervosa and anorexia) and T2DM, thus further research is needed to explore this. We also did not include articles that were not in English due to limited resources.

## Conclusion

This systematic review highlighted that people with T2DM experienced eating disorders, specifically BED and NES. There was considerable variation in the prevalence of eating disorders among the included studies. Having eating disorders in T2DM contributes to a wide range of health issues, including low levels of emotional wellbeing, body dissatisfaction, and high levels of HbA1c. Regular screening for both eating disorders in people with T2DM, along with assessing the screening results and discussing eating disorders treatment options relevant to the patient's overall health, can be essential in minimizing adverse health outcomes. Thus, healthcare professionals need to recognise these issues to improve the health of individuals with T2DM and long-term diabetes related complications. Clinicians should also be aware of the depression symptoms and anxiety associated with this disorder and recognize that people with T2DM may be hesitant to openly discuss their symptoms. There appears to be a lack of published research in this population. We emphasise the importance of large, high-quality research to better understand the management, diagnosis, and long-term outcomes of binge eating disorder in individuals with T2DM.

## Supplementary Information

Additional file 1.

## Data Availability

No datasets were generated or analysed during the current study.

## Abbreviations

T2DM: Type 2 diabetes mellitus
EDs: Eating disorders
HbA1c: Haemoglobin A1c
NES: Night eating syndrome
BES: Binge eating disorders
BMI: Body mass index
PRISMA: Preferred Reporting Items for Systematic Reviews and Meta-Analyses
SWiM: Synthesis without meta-analysis
EAT-26: Eating Attitude Test-26
QEWP-R: Questionnaire of Eating and Weight Patterns-Revised
EDE-Q: Eating Disorder Examination-Questionnaire
QEWP-26: Questionnaire of Eating and Weight Patterns
TFEQ: Three-Factor Eating Questionnaire
NEQ: Night Eating Questionnaire
