# Supplementary material for: Prevalence and associated factors of eating disorders in adults with type 2 diabetes: a systematic review
Source: J Eat Disord. 2025 Sep 26;13:211. doi: 10.1186/s40337-025-01391-y (PMC12465268; doi:10.1186/s40337-025-01391-y)
Supplement: Supplementary file 1 — Additional file 1. [file 40337_2025_1391_MOESM1_ESM.docx]

**Table 1. Medline search strategy**

| Ovid MEDLINE(R) ALL <1946 to August 28, 2024>   1. exp Diabetes Mellitus, Type 2/ 2. (non insulin$ depend$ or noninsulin$ depend$ or non-insulin$ depend$).tw,ot. 3. (t2d or t2dm or niddm or (non*1 adj insulin adj diabet*)).mp.] 4. type II [diabetes.mp](http://diabetes.mp/). 5. 1 or 2 or 3 or 4 6. exp "Feeding and Eating Disorders"/ 7. (eating disorder or anorexia nervosa or binge eating disorder or bulimia or food addiction).mp. 8. ((eating adj3 disorder*1) or (food adj addict*) or (bulimia* or bulimic* or anorexia* or anorectic*) or (binge* or binging or purg* or overeat*) or (compulsive adj3 (eat* or vomit*)) or (self*1 adj induce* adj vomit*) or (loss*1 adj control*1 adj eat*)).ti,ab. 9. 6 or 7 or 8 10. 5 and 9 11. limit 10 to (humans and "all adult (19 plus years)") |
| --- |

**Table 2. Quality assessment of included studies**

| Authors and year of publication | Quality rating (good/fair/poor) | Quality appraisal findings |
| --- | --- | --- |
| Allison, Crow [14]  2007 | Fair | Cross-sectional study  Sample size was not justified  Validity of measures was not reported |
| Celik, Kayar [32]  2015 | Fair | Cross-sectional study  Sample size was not justified |
| Crow, Kendall [37]  2000 | Poor | Cross-sectional study  Sample justification is not clear  Sample size was not justified and small sample size < 300, high risk of bias  Age range of the participants was not provided  Validity of measures was not reported |
| Herbozo, Flynn [35]  2015 | Good | Cross-sectional study  Sample size was not justified but the study includes 387 participants |
| Hood, Reutrakul [16]  2014 | Poor | Cross-sectional study  Sample size was not justified and Small sample size < 300, high risk of bias  Validity of measures was not reported |
| Kenardy, Mensch [39]  2001 | Poor | Cross-sectional study  Sample size was not justified and Small sample size < 300, high risk of bias |
| Krishnamurthy, Gupta [33]  2020 | Fair | Cross-sectional study  Participation rate and sample size was not justified  Validity of measures was not reported |
| Kumar, Alam [8]  2023 | Poor | Cross-sectional study  Participation rate and sample size was not justified  Small sample size < 300, high risk of bias  Validity of measures was not reported |
| Mannucci, Tesi [36]  2002 | Fair | Cross-sectional study  Small sample size < 300, high risk of bias |
| Muley, Deshmane [38]  2024 | Fair | Cross-sectional study  Small sample size < 300, high risk of bias |
| Nicolau, Simó [24]  2015 | Fair | Cross-sectional study  Participation rate and sample size was not justified |
| Petroni, Barbanti [34]  2019 | Poor | Cross-sectional study  Participation rate and sample size was not justified  Validity of measures was not reported |
